# Comparison of different CT metal artifact reduction strategies for standard titanium and carbon‐fiber reinforced polymer implants in sheep cadavers

**DOI:** 10.1186/s12880-021-00554-y

**Published:** 2021-02-15

**Authors:** Florian A. Huber, Kai Sprengel, Lydia Müller, Laura C. Graf, Georg Osterhoff, Roman Guggenberger

**Affiliations:** 1 Institute of Diagnostic and Interventional Radiology, University Hospital Zurich, Faculty of Medicine, University of Zurich, Raemistrasse 100, 8091 Zurich, Switzerland; 2 Department of Trauma, University Hospital Zurich, Faculty of Medicine, University of Zurich, 8091 Zurich, Switzerland; 3 Department of Orthopaedics, Trauma and Plastic Surgery, University Hospital Leipzig, 04103 Leipzig, Germany

**Keywords:** Artifacts, PEEK, Multidetector computed tomography, Image reconstruction, Diagnostic imaging, Pedicle screws

## Abstract

**Background:**

CT artifacts induced by orthopedic implants can limit image quality and diagnostic yield. As a number of different strategies to reduce artifact extent exist, the aim of this study was to systematically compare ex vivo the impact of different CT metal artifact reduction (MAR) strategies on spine implants made of either standard titanium or carbon-fiber-reinforced-polyetheretherketone (CFR-PEEK).

**Methods:**

Spine surgeons fluoroscopically-guided prepared six sheep spine cadavers with pedicle screws and rods of either titanium or CFR-PEEK. Samples were subjected to single- and dual-energy (DE) CT-imaging. Different tube voltages (80, DE mixed, 120 and tin-filtered 150 kVp) at comparable radiation dose and iterative reconstruction versus monoenergetic extrapolation (ME) techniques were compared. Also, the influence of image reconstruction kernels (soft vs. bone tissue) was investigated. Qualitative (Likert scores) and quantitative parameters (attenuation changes induced by implant artifact, implant diameter and image noise) were evaluated by two independent radiologists. Artifact degree of different MAR-strategies and implant materials were compared by multiple ANOVA analysis.

**Results:**

CFR-PEEK implants induced markedly less artifacts than standard titanium implants (*p* < .001). This effect was substantially larger than any other tested MAR technique. Reconstruction algorithms had small impact in CFR-PEEK implants and differed significantly in MAR efficiency (*p* < .001) with best MAR performance for DECT ME 130 keV (bone kernel). Significant differences in image noise between reconstruction kernels were seen (*p* < .001) with minor impact on artifact degree.

**Conclusions:**

CFR-PEEK spine implants induce significantly less artifacts than standard titanium compositions with higher MAR efficiency than any alternate scanning or image reconstruction strategy. DECT ME 130 keV image reconstructions showed least metal artifacts. Reconstruction kernels primarily modulate image noise with minor impact on artifact degree.

## Background

Orthopedic spine implants can induce CT artifacts that lead to impaired target and adjacent tissue visibility [1] with reduced image quality and eventually diagnostic yield [2]. Beyond technical issues, an increasing number of artifact corrupted CT scans can be expected in daily practice due to demographic changes that lead to a growing proportion of elderly patients with metal hardware in place [3, 4].

Metal artifacts in CT imaging occur when polychromatic energetic X-ray photons pass through dense objects, e.g. orthopedic implants. This causes comparably higher attenuation of low energy photons, i.e. photon starvation and beam hardening leading to often severe artifacts in large volume areas around the upper or lower trunk [5]. Additionally, dark streaking bands adjacent to hyperattenuating objects as well as false-bright areas imitating high attenuation tissue can appear and thus hamper diagnostic accuracy [6].

Different strategies for metal artifact reduction (MAR) in CT imaging have been proposed. On the one hand scan parameters can be changed, e.g. tube voltage to increase photon energy and decrease image noise, yet with increased radiation dose. In addition, reconstruction parameters can be modified, e.g. by using monoenergetic extrapolations (ME) with dual-energy (DE) CT or iterative reconstruction (IR) MAR techniques in single-energy (SE) scans or a combination of both [7–11]. On the other hand, substantial MAR can be achieved by optimizing metal hardware geometry and material. While standard titanium alloys are usually associated with marked artifacts, recent carbon-fiber-reinforced polyetheretherketone (CFR-PEEK) implants, usually with thin titanium shells for guidance during fluoroscopic placement only, have been shown not only to provide favorable biomechanical behavior for earlier fracture healing but also to markedly reduce metal artifacts in cross-sectional imaging [12]. While there are many studies dealing with MAR efficiency of different scanning and reconstruction techniques, the effect of recent implant hardware material on those MAR strategies has not been assessed so far.

The purpose of this ex-vivo study was to compare the MAR efficiency of different established CT scan and reconstruction strategies and to evaluate their impact on different hardware materials, i.e. standard titanium vs. novel CFR-PEEK implants of the spine.

## Methods

### Specimen

Six fresh-frozen cadavers of the thoracolumbar spine and paraspinal compartments of mature female swiss alpine sheep (AO institute, Davos, Switzerland) were warmed at room temperature and immediately processed after being completely thawed. The specimen were remainders of other biomechanical studies. No animal tissue was used for this study exclusively, therefore approval by the responsible ethics committee was waived. All fresh-frozen cadavers were known to originate from healthy animals.

### Implantation

**Fig. 1:**
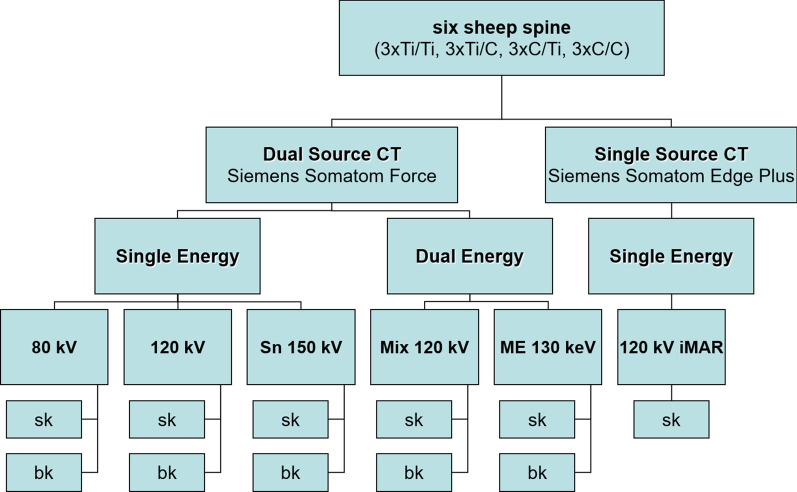
Overview of imaging workflow. All six cadaver configurations were subjected to the same imaging methods, resulting in a total of 11 image reconstructions per phantom to assess. (*CT* computed tomography, *Ti* Titanium, *C* carbon, *Sn* tin-filtered, *ME* Monoenergetic extrapolation, *iMAR* iterative metal artifact reduction, *sk/bk* soft tissue/bone kernel)

**Fig. 2:**
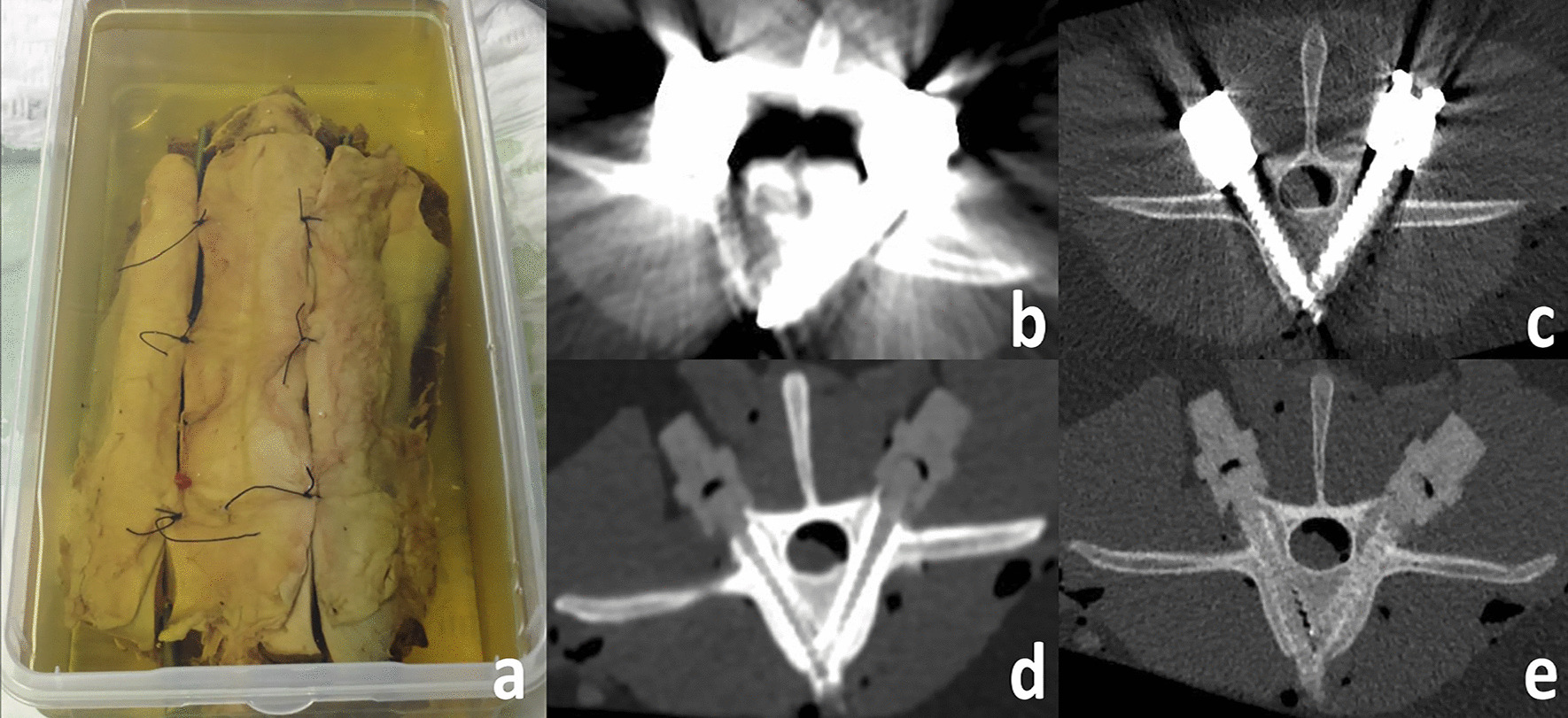
Figure **a** shows a photograph of the instrumented sheep cadavers (**a**). Impact on streak artifacts: figure **c** and **e** show representative axial images of the bestmetal artifact reduction (MAR) algorithm (bone kernel Dual Energy Monoenergetic Extrapolation 130 keV), compared to figure **b** and **d**, where the worst scans (soft kernel Single Energy 80 kV) are presented. Figure **b**, **c** show titanium (Ti/Ti) material configurations, figure **d**, **e** present a carbon configuration (C/C)

Two board-certified institute-own surgeons, specialized in spine surgery, fluoroscopically-guided implanted pedicle screws at 4 lumbar levels (L1–L5, sparing L3) bilaterally into each of the six cadavers using a clinically routinely used postero-lateral approach. Two same-sized groups (3 sheep spine each) were instrumented with FDA-approved screws either from titanium (Ti, diameter: 5.5 mm; Legacy 5.5, Medtronic Int., Tolochenaz, Switzerland) or from CFR-PEEK (C) with titanium shells (diameter 5.5 mm; CarboClear, Carbofix Orthopedic Ltd., Herzeliya, Israel). The design of the spine samples allowed to connect the screws with removable rods, made from either Ti or C (diameter 5 and 6 mm). Hence, each of the six cadaver spine specimen was assembled in two configurations depending on the removable rod-material in place. Eventually, four same-sized groups characterized by pairing of screw/rod-material were formed (C/C, C/Ti, Ti/Ti, Ti/C) and subjected to further imaging (Fig. 1). The spine samples were placed in a plastic container filled with rapeseed oil to simulate fat tissue-equivalent attenuation around the spine (Fig. 2a).

### Imaging

For the dual-source DECT scans (Somatom Force, Siemens Healthineers, Erlangen, Germany), SE-images at respective low and high tube voltages (80kVp and tin (Sn) filtered 150 kVp), as well as DE mixed images at balanced weighting of both tube voltages were generated (DE 120 kVp Mix). From DECT data, MEs were reconstructed at 130 keV (DE ME 130 keV), based on prior studies indicating best performance for different materials and hardware [13]. In addition, standard polychromatic SE images at 120 kVp (SE 120 kVp) were acquired on the DECT scanner. Secondly, specimens were scanned with the single-source scanner (Somatom Edge Plus), also using a 120 kVp protocol but with an IR MAR-algorithm (iMAR Spine, also Siemens Healthineers). Radiation dose (in CTDIvol) was matched between scan protocols in order to exclude dose-dependent effects on MAR (see Table 1). All scans were reconstructed axially with equal parameters (see Table 1) and sent to the PACS software (Impax, 6.7.0.1071, Agfa Healthcare) of our radiology department.

**Table 1:** Acquisition and reconstruction parameters. Acquisition and reconstruction parameters of all CT scans

	Dual-source CT					Single-source CT
Scan	DE				SE	SE
	80 kV	ME 130 keV	Sn 150 kV	120 kV Mix	120 kV	120 kV iMAR
Kernels (sk/bk)	Qr36/Qr54	Br36/Br54	Qr36/Qr54	Qr36d/Qr54d	Qr36/Qr54	I30s (sk)
mAs	37–47/29–33 (80/Sn 150 kV)				29–31	31–33
CTDI_vol_ (mGy)	1.81 ± 0.12				2.01 ± 0.06	2.16 ± 0.05
Slice thickness/increment	1 mm / 1 mm					
Field of view	160 × 160 mm					
Matrix	512 × 512					

*bk* bone kernel, *CT* computed tomography, *CTDI* CT dose index, *DE* dual energy, *iMAR* iterative metal artifact reduction (brand name), *ME* monoenergetic extrapolation, *SE* single energy, *sk* soft tissue kernel, *Sn* tin-filtered

### Qualitative reading

One junior and one senior radiologist (XX, YY, with 2 and 13 years of experience) interpreted all images by rating degrees of six different qualitative criteria: geometric distortion, screw-bone-interface visibility, hardware integrity, interrod-area visibility, correct screw placement and artifact penumbra of rod (between screw segments) on a four-point Likert scale (visibility: 0 = perfect, 1 = slightly reduced, 2 = severely reduced, 3 = non-diagnostic) [14]. Likert ratings were then summed up for a total score, with a potential maximum score of 6 × 3 = 18. The readers were blinded to each other as well as to materials in use and read images in random order. Viewing presets at bone window (width (W):1500/level (L):450) were kept constant for both kernels for readout.

### Quantitative reading

The same readers measured tulip and shaft diameters of each screw by a ruler tool of the PACS software. Rod diameters were measured at inter-screw-segments at vertebral disc levels in order to exclude artifact-interference from screw material. Measurements were then compared to true diameters given by manufacturer as reference standard. Additionally, HU values were measured for visually most pronounced streak artifacts neatly respecting streak borders when placing ROIs. Streaks were measured in muscle tissue adjacent to screw shaft, screw tulip and rod, and at levels analogously to sites of qualitative ratings and diameter measurements. For bone and muscle tissue reference attenuation, mean and standard deviation (SD) of HU values were measured at mid-L3-level in same-sized regions of interest (ROI). A quantitative measure of degree of streak artifacts (delta, Δ) was defined, representing differences in mean HU (ΔHU) of most pronounced streak artifacts and respective reference tissue values.

### Statistical analysis

Interrater agreement of qualitative variables was calculated with Cohen’s Kappa (κ), interreader-agreement of all quantitative parameters was interpreted with intraclass correlation coefficients (ICC). Levels of agreement were interpreted as moderate (0.41–0.60), substantial (0.61–0.80) and excellent (0.81–1.0) [15]. Data of the senior reader were used for ensuing analysis. MANOVA was performed for comparison of differences in qualitative and quantitative ratings among material compositions and scan/reconstruction-algorithms as well as radiation dose among protocols. Spearman rank-analysis was performed to test for tube-voltage and mean tissue attenuation correlation. Paired samples t-test and Wilcoxon signed-rank testing were performed for comparison of qualitative and quantitative parameters between reconstruction kernels (bone kernel and soft tissue kernel; bk and sk).

Ultimately, respective effects of MAR on pure C- and Ti-material configurations was investigated by comparison of the range of tulip and shaft diameters between overall worst and best MAR reconstruction algorithms, calculating a respective delta of the diameters (Δcm).

Post-hoc Bonferroni corrections for multiple comparisons were applied. A *p *value of < 0.05 was considered statistically significant. All calculations were performed with SPSS (v.25, IBM, Armonk, NY, USA). Figures were postprocessed with programs of the Adobe Creative Cloud (release CC 2019, Adobe Systems, San José, CA, USA).The datasets used and/or analyzed during the current study are available from the corresponding author on reasonable request.

## Results

### Qualitative parameters

Means and SD values of all cumulative qualitative measures of image quality grouped by scans and reconstruction parameters and by implant materials are listed in Table 2. Intrareader agreement over all qualitative ratings was almost perfect (κ = 0.807).

**Table 2:** Comparison of qualitative ratings. Mean cumulative values and respective standard deviations of all qualitative ratings among different reconstruction algorithms, sub-grouped by implant material configurations (screw/rod). Note significantly lower scores, i.e. higher image quality for carbon (C) screw compared to titanium (Ti) screw containing configurations. Rod material had comparably lower impact on image quality

	Ti/Ti	Ti/C	C/C	C/Ti
sk SE 80 kV	16.3 ± 0.58	15 ± 0	1.08 ± 0.07	5.54 ± 1.53
bk SE 80 kV	16.3 ± 0.58	15 ± 0	1 ± 0.13	5.50 ± 1.42
sk SE 120 kV	16.3 ± 0.58	14.92 ± 0.14	0.75 ± 0.33	4.6 ± 0.19
bk SE 120 kV	16 ± 0	15 ± 0	0.5 ± 0.71	3.53 ± 0.30
sk SE 120 kV iMAR	15.36 ± 0.13	12.46 ± 2.15	0.42 ± 0.14	2.7 ± 0.17
sk DE 120 kV Mix	12.97 ± 2.71	12.21 ± 2.55	0.25 ± 0.25	2.10 ± 0.95
bk DE 120 kV Mix	12.63 ± 3.07	12.25 ± 2.54	0 ± 0	1.97 ± 0.91
sk SE Sn 150 kV	10.65 ± 0.65	10.21 ± 0.36	0 ± 0	0.53 ± 0.50
bk SE Sn 150 kV	10.35 ± 0.60	10.21 ± 0.36	0 ± 0	0.3 ± 0.3
sk DE ME 130 keV	10.25 ± 0.66	9.3 ± 0.63	0 ± 0	0 ± 0
bk DE ME 130 keV	9.92 ± 0.14	9.08 ± 0.80	0 ± 0	0 ± 0

*bk* bone kernel, *C* carbon, *DE* dual energy, *iMAR* iterative metal artifact reduction (brand name), *ME* monoenergetic extrapolation, *SE* single energy, *sk* soft tissue kernel, *Sn* tin-filtered, *Ti* titanium

### Impact of implant material on artifact degree

**Fig. 3:**
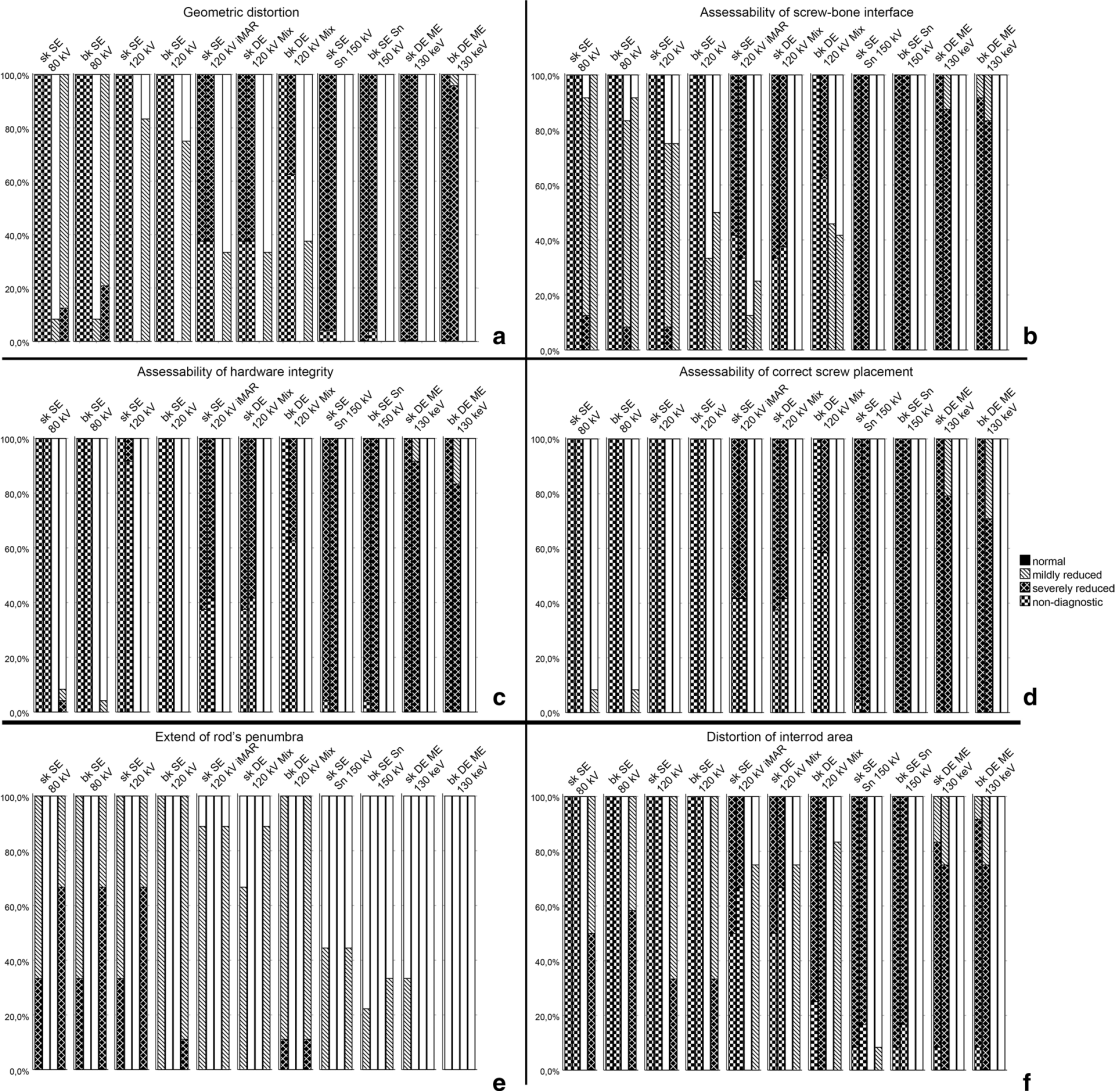
Distribution of Likert scale ratings (0 = perfect visibility, 1 = mildly reduced visibility, 2 = severely reduced visibility, 3 = non-diagnostic) for all six qualitative parameters (**a**–**f**), among reconstructions and material configurations. Neighboring columns show values for the four different material configurations of carbon and titanium (C and Ti, from left to right: Ti/Ti, Ti/C, C/C and C/Ti for screws and rods, respectively)

Significant differences in qualitative ratings were seen among both implant materials and scan/reconstruction-methods with a significantly larger impact of the first (F = 1562 vs. 39, both *p* < .001, Fig. 3). Cumulative values of the six Likert-rated categories for C-screw were markedly lower, i.e. better than Ti-screw containing configurations, ranging overall from 0.33 ± 0.44 (C/C) to 13.65 ± 2.91 (Ti/Ti) of a potential maximum score of 18. The C/C configuration ranked always significantly better compared to material configurations containing either Ti screws or rods (all *p* < .001).

#### Impact of image reconstruction and MAR strategy on artifact degree

Image reconstruction and MAR strategies had a marked impact on artifacts from Ti-implants but were almost negligible in artifact poor C-implants. Bk DE ME 130 keV showed best cumulative qualitative ratings of all images (4.75 ± 4.98) being significantly lower (*p* < .001) than the remainder, except for sk DE ME 130 keV and SE Sn 150 kV images (*p* = 1). The worst ratings were found for sk SE 80 kV, being significantly higher (*p* < .01) than the remainder. Sk SE 120 kV iMAR images performed better than standard sk or bk SE 120 kV images (*p* = .068) but markedly inferior to any SE Sn 150 kV or DE ME 130 keV reconstruction (*p* < .001).

Despite slightly lower, i.e. better scores for bk reconstructions, there were no significant overall differences between bk and sk, respectively (z = −0.943, *p* = .345).

### Quantitative parameters

The distribution of means and SDs of all quantitative parameters, i.e. ΔHU and reference tissue attenuation values are given in Table 3. Inter-reader agreement was perfect for both types of quantitative parameters, with ICCs of 0.974 (HU) and 0.967 (diameters).

**Table 3:** Distribution of streak artifacts and noise among different reconstruction algorithms and implant configurations (screw/rod). Distribution of most pronounced streak artifacts of the screw shaft (in ΔHU) and corresponding reference HU values of bone and fat tissue among different image reconstruction algorithms. Noise was measured as ± standard deviation (SD) of reference muscle attenuation. Note significantly lower artifact degrees for carbon (C) screw compared to titanium (Ti) screw containing configurations. Rod material had comparably lower impact on artifact degree. Mean HU-values of reference muscle tissue showed no significant differences among reconstructions-algorithms (F = 0.815, *p* = .615) while in the vertebral body significant inverse correlation with tube voltage (Spearman’s rho= −656, *p* < .001) was seen. Image noise inversely correlated with tube voltage

	Artifact degree (ΔHU) of screw shaft				Reference tissue attenuation (HU)		
	Ti/Ti	Ti/C	C/C	C/Ti	Bone	Muscle	Noise (± SD)
sk SE 80 kV	− 859	− 903	− 37	− 32	872	63	19
bk SE 80 kV	− 870	− 898	− 35	− 58	871	63	45
sk SE 120 kV	− 848	− 897	− 11	− 39	670	60	14
bk SE 120 kV	− 876	− 920	− 48	− 69	656	61	37
sk SE 120 kV iMAR	− 768	− 719	− 71	− 106	628	59	16
sk DE 120 kV Mix	− 664	− 630	1	− 58	559	61	15
bk DE 120 kV Mix	− 713	− 674	− 1	− 63	562	61	43
sk SE Sn 150 kV	− 648	− 617	0	− 7	474	60	17
bk SE Sn 150 kV	− 691	− 641	− 10	− 5	477	60	53
sk DE ME 130 keV	− 615	− 582	13	− 17	402	59	18
bk DE ME 130 keV	− 690	− 608	11	− 25	401	60	46

*bk* bone kernel, *C* carbon, *DE* dual energy, *HU* Hounsfield Units, *iMAR* iterative metal artifact reduction (brand name), *ME* monoenergetic extrapolation, *SD* standard deviation, *SE* single energy, *sk* soft tissue kernel, *Sn* tin-filtered, *Ti* titanium

#### Impact of implant material on artifact degree

**Fig. 4:**
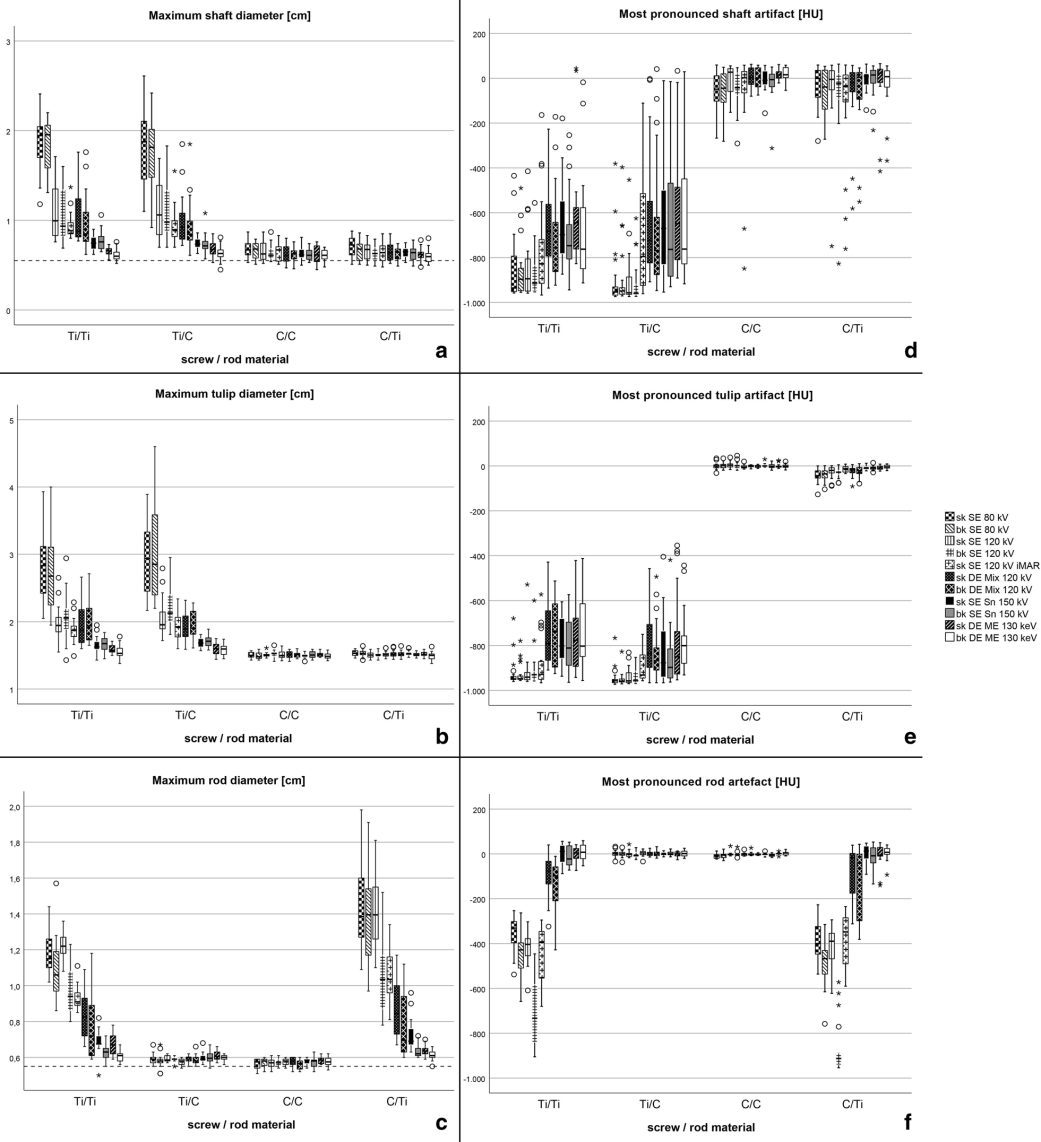
Diameters (**a**–**c**, left column) and ΔHU (**d**–**f**, right column) of shaft, tulip and rod, respectively, as quantitative measures of artifacts. All values are separated per reconstruction and per material configuration in use (similar to Fig. 2). Dashed lines indicate true dimensions of shafts and rods as given by manufacturer

The distribution of means and SDs of all quantitative parameters, i.e. ΔHU and screw/rod-diameters was significantly different among implant materials (F = 1744 and 462, all *p* < .001) and scan/reconstruction-algorithms (F = 18 and 122, all *p* < .001) with a substantially larger impact of the first than the latter on artifact degree (Fig. 4; Table 3**)**. C-material derived diameter measurements were significantly closer to true dimensions than Ti-materials, independent of measurement site (screw-tulip, -shaft and rod) (Fig. 4).

#### Impact of image reconstruction and MAR strategy on artifact degree

**Fig. 5:**
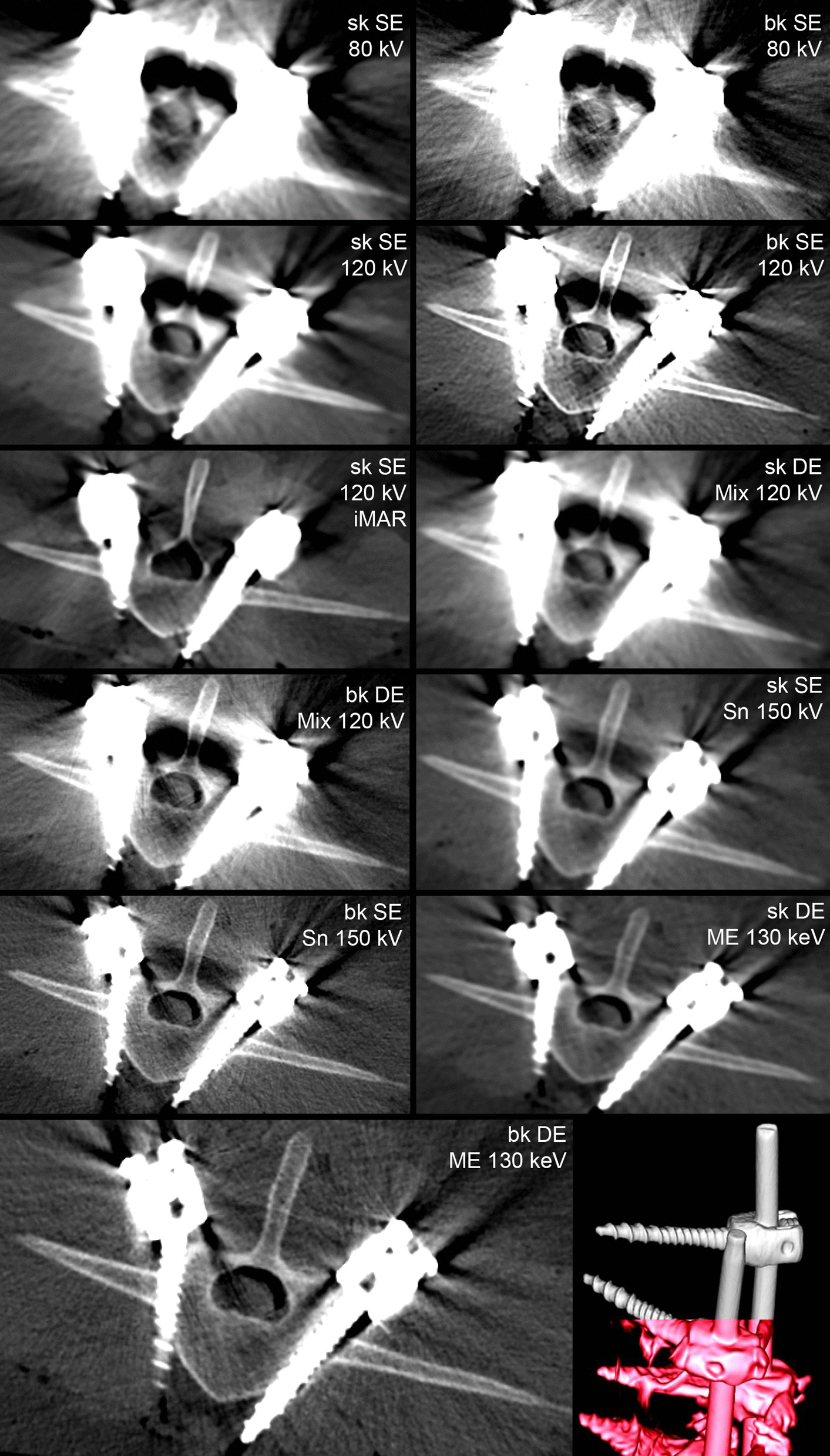
Comparison of energy levels in order of increasing overall qualitative ratings from left to right and from top to bottom, respectively. Representative images show an exemplary sample with Ti/Ti composition of screws and rods. The bottom right 3D volume-rendered reconstruction is a composite image of most pronounced streak artifacts in the best (upper half) and worst (bottom half) metal artifact reduction (MAR) algorithm, respectively

Comparing image reconstruction and MAR strategies, significant overall differences in ΔHU of the screw-shaft and -tulip as well as rod artifacts were found (*p* < .05) and as expected ΔHU increased (i.e. less artifacts) with increasing tube voltage (see Fig. 5). Sk SE 120 kV iMAR performed better than standard SE 120 kV images, but inferior to SE Sn150 kV and DE ME 130 keV with least artifacts. For all diameter measurements, DE ME 130 keV- and SE Sn 150 kV-derived measurements fitted best true diameters of the implants, differing significantly from the remainder (*p* < .001) but not from each other.

No significant overall differences of ΔHU and diameter measurements of screws were found between different kernels (bk and sk; *p* = .102 and 0.525) regardless of materials. However, rod ΔHU was significantly smaller in sk versus bk (−107.02 ± 180.40 vs. −184.31 ± 312.22, *p* < .001) and diameter measurements were significantly closer to real dimensions in bk versus sk (0.777 ± 0.286 vs. 0.738 ± 0.257, *p* < .05) (Fig. 3).

With respect to impact of implant material on MAR efficiency, the diameter difference of streak artifact between worst (sk SE 80 kV) and best (bk DE ME 130 keV) MAR strategy was compared between C/C- and Ti/Ti-configurations. Δcm was significantly smaller in pure C/C as compared to Ti/Ti compositions for both tulip (0.01 ± 0.05 vs. 1.29 ± 0.45) as well as for shaft (0.08 ± 0.07 vs. 1.26 ± 0.30, both *p* < .001) measurements.

Mean HU-values of reference muscle tissue showed no significant differences among scan/reconstructions-algorithms (F = 0.815, *p* = .615) while in the vertebral body significant inverse correlation with tube voltage (Spearman’s rho= −656, *p* < .001) was seen (Table 3**)**.

Despite matched radiation dose between scan protocols [mean CTDIvol of 1.81, 2.01 and 2.16 mGy for DECT and both SECT scans (SE 120 kV and SE 120 kV iMAR)], slight but significant dose differences (*p* < .001) were seen but within 10 % range of total dose.

## Discussion

This study investigated the effect of different MAR-strategies in CT-imaging of spine implants and compared their efficacy in different hardware materials ex vivo using dedicated sheep cadavers.

The most efficient MAR strategy was to use CFR-PEEK as spine implant material. This was seen both in qualitative and quantitative artifact measures, where the worst scan/reconstruction-algorithm for C/C-configurations achieved significantly higher diagnostic quality than the best performing reconstructions (DECT MEs) for Ti/Ti-compositions (see Fig. 2b**/**c). Thus, the significant impact of recent CFR-PEEK implants with respect to artifact degree by far outweighs technical innovations to reduce metal artifacts. In addition to substantial MAR [12], CFR-PEEK implants have also been shown to offer good biocompatibility and osseointegrative behavior [16–18] further advocating an increasing role in spine surgery.

On the other hand, we demonstrated the essential value of reconstruction-based MAR for standard Ti/Ti-compositions, to date still the material being most frequently used for orthopedic hardware. Especially implant diameter measurements in Ti implants showed significantly larger variations among reconstruction algorithms as compared to C/C-compositions. This should be considered whenever CFR-PEEK configurations are not available. Despite comparable costs of both implant materials, there may be restrictions in certain countries on the use of CFR-PEEK implants for only defined indications (i.e. in patients where stereotactic radiation therapy or frequent imaging follow-ups are planned). In CFR-PEEK however, the impact of advanced MAR strategies was almost negligible. Considering higher costs of DECT scanners and MAR-software as compared to standard single energy CT scanners and the broader availability of the latter, this may further add to the increasing popularity of carbon implants.

Beside the major factor of hardware material, significant differences in MAR efficiency were also detected among different MAR reconstruction strategies. Differences were much more pronounced in Ti-containing compositions, especially around Ti-screw shafts and almost diminished with C-implants. This largely conforms with a series of studies that have demonstrated the efficacy of different MAR-techniques in imaging of traditional hardware [7, 19–21]. Increasing tube voltage and thus beam energy generally leads to less image noise and metal artifacts. This is reflected in our data, where Sn150 kV-images showed less artifacts than low energy 80 kV-images from DECT scans and standard SE 120 kV reference images. As shown in various studies, efficient MAR can also be achieved by both MEs in DECT, as well as IR-technique in SECT imaging (e.g. iMAR) or a combination of both [7, 11, 22, 23]. Our results concur with these findings demonstrating significant MAR for both approaches, with better qualitative and quantitative data for DECT ME 130 keV. The fact that at the time when this study was conducted the iMAR software could only be applied to soft tissue kernels may have further biased this comparison. We did not include a combination of DECT-based ME and IR-techniques in our analysis as there are conflicting results about its benefit [10, 11]. Current literature favors IR-techniques due to ease of use (no manual ME reconstruction), larger applicability (SECT scanners more frequent), price of scanner-unit and better comparability with other institutes [11, 24, 25]. Yet, our data showed excellent homogeneity of reference muscle tissue attenuation among different scanners and MAR strategies reflecting the robustness of the different approaches.

The appearance of metal artifacts significantly changes between viewing windows but the respective influence of reconstruction kernels, e.g. sk versus bk cannot be simply inferred and was hence further investigated in this study. In order to exclude viewing-associated factors, predefined standard window settings (W:1500/L:450) were used for readout. As expected, a significantly higher image noise was measured in bks and a tendency of more pronounced shaft and rod artifacts from Ti-implants in bk than sk images without significant impact on artifact degree was seen. On the other hand, true shaft and rod dimensions in Ti-implants were better approximated in bk compared to sk. Despite sharper and more accurate depiction of implants, artifacts from Ti-components are slightly accentuated by bk reconstructions while artifacts from CFR-PEEK-implants remain largely unchanged by different kernels. Hence, bk images may be a better option to look for implant material wear or fracture, while peri-implant osteolysis or soft tissue pathology may be better visible on sk images.

There are limitations to this ex vivo study as we did not assess artifacts in vivo. However, the use of sheep cadaver allowed for repeated scans with standardized acquisition and reconstruction protocols. Thus, the validity for in-vivo conditions can be largely inferred. Due to different scanner designs (single-source vs. dual-source CT) absolute dose standardization could not be obtained. However, image noise as a sensitive indicator of radiation dose variations did not significantly differ among 120 kV-images from different scanners (SE 120 kV, DE 120 kV Mix, SE 120 kV iMAR). IR-software was only applicable to sk 120 kV images. According to recent publications [10, 11], this may become obsolete in the near future further increasing the popularity of IR techniques. Furthermore, we focused on common IR-strategies, but did not comment on recent innovations in that field, e.g. model-based IR [26]. Lastly, we have compared CFR-PEEK to standard Ti-implant material only. Different designs and alloys may show different behavior in MAR strategies. However, the significant advantage in MAR of C-based vs. mere metal implants as shown in this study may remain largely independent of metal type.

## Conclusions

In conclusion, titanium-shell CFR-PEEK implants induce significantly less artifacts than standard Ti-compositions. This effect is by far stronger than any other MAR strategy. DECT ME 130 keV achieved best MAR while reconstruction kernels modulate image noise with minor impact on artifact degree. MAR reconstruction strategies may be negligible for CFR-PEEK implants but are essential for standard metal implants.

## Data Availability

The datasets used and/or analysed during the current study are available from the corresponding author on request.

## Abbreviations

bk: Bone kernel
C: Carbon
CFR: Carbon-fiber-reinforced
CT: Computed tomography
CTDIvol: Volume computed tomography dose index
DE: Dual energy
ICC: Intra-class correlation
IR: Iterative reconstruction
kVp: Peak kilovoltage
(M)ANOVA: (Multivariate) Analysis of variance
MAR: Metal artifact reduction
ME: Monoenergetic extrapolation
PEEK: Polyetheretherketone
SE: Single energy
sk: Soft-tissue kernel
Sn: Tin
Ti: Titanium
